# Results of Conversion from Failed Austin-Moore Hemiarthroplasty to Cementless Total Hip Arthroplasty in Octogenarian Patients with Advanced Acetabular Erosion: A Minimum of 5 Years of Follow-Up

**DOI:** 10.1155/2019/7814602

**Published:** 2019-04-02

**Authors:** Tsan-Wen Huang, Chih-Hsiang Chang, Fu-Chun Chang, Chun-Chieh Chen, Kuo-Chin Huang, Mel S. Lee, Hsin-Nung Shih

**Affiliations:** ^1^Department of Orthopaedic Surgery, Chang Gung Memorial Hospital, Chiayi, Taiwan; ^2^Chang Gung University, Taoyuan, Taiwan; ^3^Department of Orthopaedic Surgery, Chang Gung Memorial Hospital, Linkou, Taiwan; ^4^Bone and Joint Research Center, Chang Gung Memorial Hospital, Linkou, Taiwan; ^5^Department of Orthopaedic Surgery, Kaohsiung Chang Gung Memorial Hospital, Taiwan

## Abstract

Austin-Moore hemiarthroplasty (HA) had been selectively used for elderly patients with femoral neck fractures. With increasing life span and activity, the sequela of Austin-Moore HA make the implant no longer favorable. The treatment of failed Austin-Moore HA with advanced acetabular erosion is challenging; however, little has been published regarding this topic. The aim of this study was to evaluate the mid-term results of using cementless total hip arthroplasty (THA) in octogenarians. Between 2008 and 2011, 47 patients (32 women and 15 men) with an average age of 86 years (range 83-89 years) were enrolled in this retrospective study. After an average follow-up period of 6.2 years (range 5.0-7.8 years), no migration or loosening of the cup or femoral stem was found. Harris hip scores improved from 36 (range 15-42) preoperatively to 87 (range 80-90). There were no complications directly associated with the procedure except for superficial infections in two patients. Our results suggest that using cementless THA can result in favorable radiographic and clinical outcomes in octogenarian patients.

## 1. Introduction

The high morbidity and mortality rates following osteoporotic femoral neck fractures can be devastating for the patient and increase the burden on health care systems [1, 2]. Except in patients with an extremely poor health status, unipolar or bipolar hemiarthroplasty (HA) has been proposed to be a favorable procedure to provide pain relief and restoration of hip function and thereby decrease complications related to long-term confinement to bed [3–9]. The number of HA procedures is increasing accordingly [6].

Austin-Moore HA had been selectively used for elderly patients with femoral neck fractures due to its relatively low cost and good short-term results [7–9]. With an increasing life span and activity, increasing attention is being paid to the mid-term and long-term outcomes of Austin-Moore HA [10–15]. The failure rate of the Austin-Moore prosthesis has been reported to be 2-10% at mid-term follow-up and 6-35% at long-term follow-up [10–15]. Acetabular cartilage degeneration is a common complication and is a critical indication for conversion to total hip arthroplasty (THA) [6–9]. A unipolar prosthesis has the disadvantage that movement of the hip joint occurs between the metal head and the natural acetabulum. The metal-on-cartilage interface is subject to wear, which is considerably higher than with cartilage-cartilage and metal-polyethylene surfaces [11]. In addition, coexisting osteoarthritis and osteoporosis may contribute to faster destruction of acetabular cartilage and acetabular erosion in elderly patients [12, 13].

In contrast to a failed bipolar prosthesis, conversion of a unipolar prosthesis in a non-infected hip with advanced acetabular erosion is technically difficult [16]. High incidence rates of periprosthetic fractures, femoral perforation and instability have been reported during conversion to THA [16–18]. Few reports have addressed this situation or reported mid-term results [6–9]. Therefore, the purpose of this study was to describe the surgical technique and report mid-term outcomes of converted cementless THA in octogenarians with advanced acetabular erosion following Austin-Moore HA.

## 2. Materials and Methods

### 2.1. Ethics

This retrospective study was approved by the Ethics Committee and Institutional Review Board of authors' institution, and all patients provided signed informed consent.

### 2.2. Demographics

All patients who undergo revision THA at the authors' institution are routinely enrolled in our arthroplasty registry. We prospectively collected demographic data, preoperative and postoperative radiographic and clinical functional assessment data. Radiographs were reviewed to determine the grade of acetabular erosion according to the criteria of Baker et al. [19]. We manually reviewed our arthroplasty database to identify the patients who had grade 2 and grade 3 acetabular erosion and underwent converted cementless THA. To minimize surgeon-related confounding factors, all enrolled patients' index Austin-Moore HAs were performed by our senior author (Hsin-Nung Shih) and were all done using the anterolateral approach and cemented fixation. All of the converted cementless THA procedures were also performed by the same surgeon. The patients who (a) were followed for < 5 years, (b) were not ambulatory preoperatively, (c) had delirium or dementia and could not cooperate to assess the functional outcomes, and (d) had incomplete medical records, radiographic analyses, or clinical functional assessments were excluded.

### 2.3. Surgical Technique

**Figure 1:**
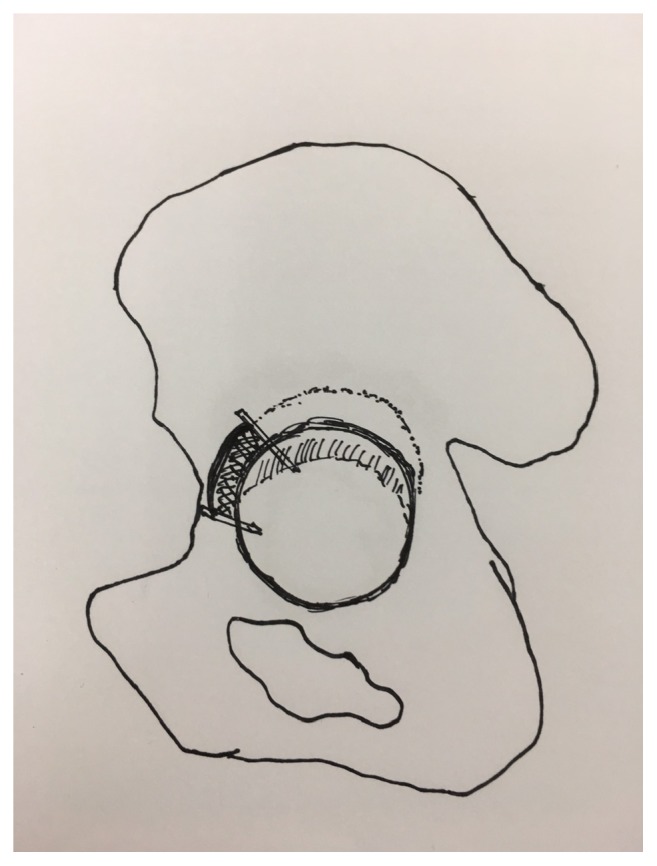
The direction of osteotomy over acetabular rim was done based on axis of prosthesis neck, and the width was the same as diameter of Austin-Moore prosthesis to dislocate the femoral head. Osteotomy over dome area should be avoided to prevent inadequate support of later acetabular shell implantation.

**Figure 2:**
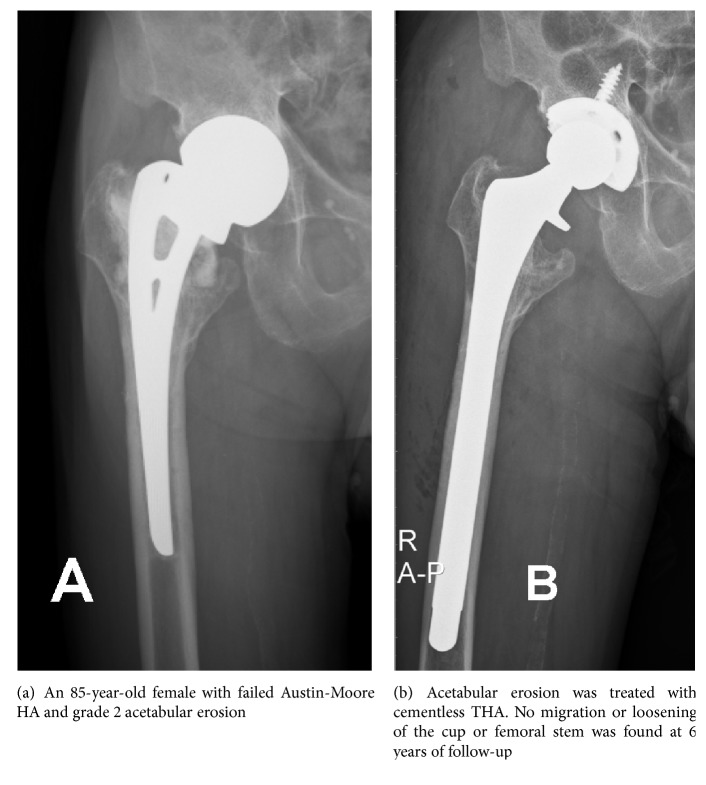


**Figure 3:**
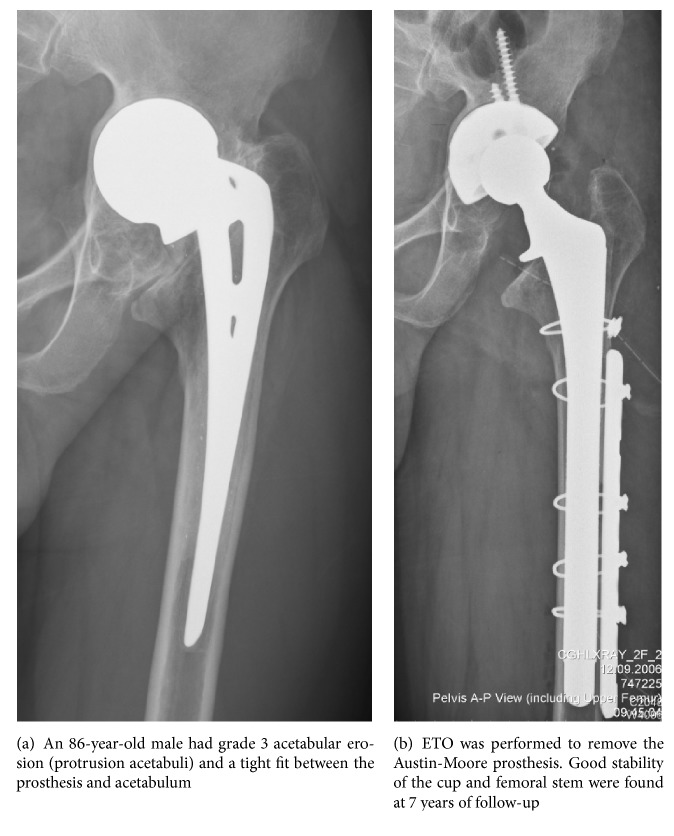


The patients were placed in the lateral decubitus position on the operating table. All operations were performed by a single senior surgeon using the anterolateral approach. In patients with advanced acetabular erosion, dislocating the large-diameter unipolar prosthesis is very challenging technically. Before dislocating the prosthesis, the scar tissue was carefully released from around the prosthesis. To prevent over release of soft tissue, we recommend a limited sacrifice of anterior rim to dislocate the prosthesis anteriorly. All of our approach is anterolateral, so the direction of osteotomy over acetabular rim was done based on axis of prosthesis neck, and the width was the same as diameter of Austin-Moore prosthesis to dislocate the femoral head. Osteotomy over dome area should be avoided to prevent inadequate support of later acetabular shell implantation (Figure 1). Care was taken to check synchronous motion of the femur with the prosthesis to avoid a periprosthetic fracture [8]. To remove the prosthesis from the femur, the femoral component can be loosened and removed simply by using a commercially available extraction device. After removing any bone that may impede stem removal and separating the proximal cement, extracting the cemented stems usually presented no major problems (Figure 2). However, in cases with protrusion acetabuli and a tight fit between the prosthesis and acetabulum, we recommend performing an extended trochanteric osteotomy (ETO) initially to extract the prosthesis from the femur and then extract the prosthetic head from the protrusion acetabuli (Figure 3). After the previous Austin-Moore prosthesis was removed, the fibrous tissue or cement mantle in the femoral canal was thoroughly debrided and removed with curettage or high-speed burring.

In protrusion acetabuli, the prosthetic head results in a medial bony defect. We treated the contained defect with a morselized allograft using an impactor and reverse reaming. In patients with a large medial wall defect, we used a witch's hat-shaped structural allograft to reconstruct the acetabulum to become a contained defect. The morselized allograft was then applied to reshape the acetabular cavity. This technique is easy and provides favorable long-term results [20]. In femoral preparation, we used an interlocking nail guide wire to avoid the false tract followed by reaming using sequentially larger cannulated flexible reamers. A full porous-coated femoral stem was used in all patients, with a length depending on the femoral bone stock. The femoral stem should be long enough to allow for endosteal cortical contact over a distance of 5 to 6 cm.

Prophylactic antibiotics were started intravenously preoperatively and were continued for 3 days after surgery. Non-weight bearing mobilization with two crutches was allowed, but weight bearing was avoided for 3 months until radiographic evidence of graft union was observed.

### 2.4. Assessment

An independent assessor blinded to the patients' demographic data performed the radiographic and clinical assessments. All patients were evaluated by radiographic analysis using antero-posterior radiographs of the pelvis and antero-posterior and lateral radiographs of the hip taken preoperatively and postoperatively. Postoperative radiographic assessments were performed after 6 weeks, 3 months, 6 months, 1 year, and then annually. Clinical assessments included American Society of Anesthesiologists classification, gait, leg length, range of motion, and neurological status. Modified Harris hip scores (HHS) were recorded preoperatively and at last follow-up. Complications such as venous thromboembolism, neurovascular injury, periprosthetic fracture, dislocation, implant malposition, implant size mismatch, and early loosening were recorded.

### 2.5. Statistical Analysis

All data were collected and entered into a Microsoft Excel spreadsheet by an independent surgeon who was blinded to the surgical techniques used. After the spreadsheets had been rechecked for missing and illogical data, the data were copied into SPSS 13.0 for Windows (SPSS Inc., Chicago, IL, USA) and analyzed. The paired* t*-test was used to compare the modified HHS recorded preoperatively with those recorded at the last follow-up assessment. All data were analyzed by an independent statistician who was blinded to the surgical outcomes. Significance was set at* p* < 0.05.

## 3. Results

Between 2008 and 2011, 79 converted arthroplasties using cementless THA prostheses for failed Austin-Moore HA with advanced acetabular erosion were performed by our senior author. Among them, 14 patients died from non-related causes, 5 patients were lost to follow-up, 2 patients were not ambulatory preoperatively, 3 patients had delirium or dementia and could not cooperate to assess the functional outcomes, and 8 patients had incomplete data. These patients were excluded. A total of 47 patients (32 women and 15 men) met our inclusion criteria. The mean age at surgery was 86 years (range, 83-89 years), and 28 patients were ASA class II and 19 were ASA class III. The mean body mass index was 26.8 kg/m^2^ (range, 21.7-39.8 kg/m^2^). The mean time between Austin-Moore HA and converted THA was 5.7 years (range, 3-7.1 years), and the mean duration of follow-up was 6.2 years (range, 5.0-7.8 years).

The mean operating time was 153 minutes (range, 110-234 minutes), the mean amount of blood loss was 522 mL (range, 300-950 mL), and the mean hospital stay was 6 days (range, 4-9 days). Radiographic evaluations showed that the mean cup inclination angle was 44.3°  ± 5.1° (range, 38°-50°) at the initial examination and 45.2°  ± 4.7° (range, 39°-50°) at last follow-up. Minor graft resorption was noted in six patients with radiolucent lines of less than 2 mm without cup migration at final follow-up. The other patients had good graft incorporation with trabecula bridging the graft and host bone. During serial follow-up, no migration or loosening of the cup or femoral stem was found.

The mean modified HHS showed significant improvements, from a mean preoperative score of 36 (range, 15-42) compared to 87 (range, 80-90) at last follow-up (*p* < 0.05). There were no deep infections, venous thromboembolism, intra-operative periprosthetic fractures, and dislocation or neurovascular complications such as sciatic nerve or peroneal palsy; however two patients developed superficial infections and received antibiotic therapy.

## 4. Discussion

The most important finding of this study is that converted cementless THA was an efficacious treatment strategy for failed Austin-Moore HA with advanced acetabular erosion in octogenarian patients. We found significant improvements in pain relief and restoration of function as well as a low complication rate at a mean follow-up of 6.2 years.

Austin-Moore HA had been selectively used for elderly patients with femoral neck fractures [3–9]. However, a poor quality of life and unsatisfactory results reportedly affect as many as 48% of physically active elderly patients [8]. The reported percentage of prosthesis survival ranges from 2% to 10% at mid-term follow-up and from 6% to 35% at long-term follow-up [10–15]. Clayer and Bruckner investigated the long-term outcomes of Austin-Moore HA and found that 31% of the patients were community ambulators, 35% household ambulators, 4% nonfunctional ambulators, and 30% nonambulators at 10 years of follow-up [10]. Furthermore, patients younger than 70 years have been reported to have a significantly higher revision rate [12]. Grosso et al. [21] reviewed the best available evidence in the literature and concluded that a cemented, unipolar HA should be reserved for low-demand elderly patients. However, the relatively low cost and good short-term outcomes have resulted in widespread use in inappropriate patients [7, 8]. With increasing life span and activity, the number of failed Austin-Moore HA procedures is increasing [6–9].

Several studies have reported favorable outcomes of converted THA for failed Austin-Moore HA [6–9]. Cossey and Goodwin reported that 88% of patients had satisfactory outcomes at 1 year of follow-up [6], and Pankaj et al. [7] reported a significant functional improvement from an HHS of 38 to 86 at an average 6.4 years of follow-up. Bhosale et al. also reported an improvement in HHS from 65 to 87 after conversion of failed Austin-Moore HA to THA [8]. However, few reports have evaluated the mid-term results of cementless THA in the treatment of advanced acetabular erosion in octogenarians [8]. In octogenarian patients, coexisting osteoarthritis and osteoporosis may contribute to faster destruction of acetabular cartilage and advanced acetabular erosion. In contrast to a bipolar prosthesis, a unipolar prosthesis is a one-piece design. When there is a tight fit between the prosthesis and acetabulum, it is very challenging technically in terms of soft tissue management and extraction of a large-diameter one-piece prosthesis without unexpected bony destruction during conversion to THA [9]. High incidence rates of periprosthetic fractures, femoral perforation, and instability have been reported during conversion to THA [8, 9, 17, 18]. In addition, restoration of acetabular bone stock is an important confounding factor, which often compromises the support of the acetabular component [20].

In their retrospective study, Bhosale et al. [8] studied 19 patients with advanced acetabular erosion and reported favorable mid-term outcomes using morselized pieces of autogenous iliac crest bone graft and cemented THA (all cemented stems with cemented cups [n=16] and anti-protrusion cages [n=3]). Because harvesting an autograft may lead to donor-site morbidity, there are legitimate concerns about using them [2, 22]. In addition, cementless THA, which has gained substantial popularity in the past decades, was developed to solve problems pertaining to cemented implants, such as toxicity of bone cement, tissue destruction by heat during polymerization, and other technical difficulties [23, 24]. In this study, 47 patients who underwent converted cementless THA using morselized allografts with or without a witch's hat-shaped structural allograft were retrospectively reviewed at a minimum follow-up period of 5 years postoperatively. At a mean follow-up period of 6.2 years, the average HHS improved from 36 preoperatively (range, 15-42) to 87 (range, 80-90) at the last follow-up. Only two (4.3%) patients developed superficial infections, and no migration or loosening of the cup or femoral stem was found at the last follow-up.

Sierra and Cabanela [9] studied 132 failed HA that were converted to THA and reported dislocations in 9.8% after a mean follow-up of 7.1 years. The soft-tissue envelope is particularly critical to the stability following conversion to THA [17, 18]. In theory, extending the soft-tissue dissection would facilitate dislocation of a large-diameter unipolar prosthesis and preserve bone stock as well as preventing periprosthetic femoral fractures. However, conversion of a HA to a THA differs from revisions of a THA, and a substantial reduction in the size of the prosthetic femoral head is unique to conversion of a HA. Subsequent improper soft-tissue tension and a wide extent of soft tissue release may contribute to postoperative dislocation following converted hip surgery [15, 16]. In this investigation, limited sacrifice of the anterior rim facilitated the removal of the endoprosthesis and prevented improper soft tissue dissection. In addition, limited sacrifice of the anterior rim would not compromise the bone stock or stability of the cementless cup component. In patient with protrusion acetabuli and a tight fit between the prosthesis and acetabulum, we recommend performing an ETO initially to extract the prosthesis from the femur and then extracting the prosthetic head from the protrusion acetabuli. No dislocation or loosening of the cup component or femoral stem was noted at a mean follow-up of 6.2 years in our series.

The main limitations of this study are that it was not prospective or randomized, and it is limited by its retrospective design, small number of cases, and lack of a control group. Failed Austin-Moore HA with advanced acetabular erosion mainly occurred in physically active elderly patients with a relatively longer life expectancy. Despite the relative rarity, we conducted a relatively large study. In addition, confounding factors may have been reduced because all of the patients were treated by the same experienced surgeon, with the same surgical technique, and with the same treatment protocol. Another limitation must be acknowledged: we did not evaluate the global health data and bone quality measurements at the proximal femur. These may have been important but both of the evaluations were not in our treatment protocol.

## 5. Conclusion

Conversion from failed Austin-Moore HA with advanced acetabular erosion to THA in octogenarians is challenging. Special attention should be given to prevent intraoperative periprosthetic fractures and instability. Our data suggest that cementless THA is an efficacious treatment strategy for such cases.

## Data Availability

The data used to support the findings of this study are available from the corresponding author upon request.
